# To study the intervention mechanism of pediatric massage on intestinal flora and host metabolism in children with anorexia

**DOI:** 10.1097/MD.0000000000023349

**Published:** 2020-11-20

**Authors:** Hanyuan Gao, Xutong Zhang, Wenjie He, Xia Zhao, Juan Han, Dongmei Li, Hanteng Yang, Shengcai Li

**Affiliations:** a Gansu Provincial Hospital of Traditional Chinese Medicine; b Gansu University of Chinese Medicine; c Gansu Institute of Chinese Medicine; d Lanzhou University Second Hospital, Lanzhou, Gansu, China.

**Keywords:** child massage, clinical trial protocol, intestinal flora, pediatric anorexia

## Abstract

**Background:**

As a common and frequent disease in pediatric patients, pediatric anorexia (PN) poses a serious threat to childhood growth and health. In recent years, societal changes in lifestyle and diet have increased the incidence of this PN, which has attracted extensive attention from both the medical community and parents. It has been shown that massage therapy represents an effective intervention for the treatment of anorexia, but investigation on its mechanism(s) of action remains limited. In this study, we will explore the biological mechanism(s) of PN from the perspective of intestinal flora, to further reveal its site of action and therapeutic mechanism(s).

**Methods:**

A total of 60 healthy children will be randomly selected for physical examination. According to a random number generated by a computer, children with anorexia who meet the inclusion criteria will be selected. In strict accordance with the time sequence of inclusion, subjects will be randomly assigned to either the massage or control group (n = 60 per group). The blank group will receive no treatment. Children in the massage group will receive a designated massage protocol. The control group will be administered oral Jianweixiaoshi tablets over 4 weeks. Each group will be compared for intestinal flora structure, fecal short chain fatty acids levels, serum trace elements, urine D-xylose-excretion rates, gastric fluid emptying, gastric motility, and hemoglobin levels before and after treatment.

**Results:**

We will review the clinical trial registry in China (http://www.chictr.org.cn/searchprojen.aspx), peer-reviewed journals and academic conferences.

**Conclusion:**

This study will verify the intervention mechanism(s) of pediatric massage on intestinal flora and host metabolism in children with anorexia.

**Trial registration number:**

ChiCTR2000033274

## 1 Introduction

Feeding disorders fall into 3 categories under the Chatoor criteria:^[1]^

(1) feeding disorders of state regulation;
(2) feeding disorders of reciprocity; and
(3) pediatric anorexia (PN).

PN refers to children with a sustained loss of appetite, who eat less and/or dislike eating. As a disorder of chronic hunger, PN is common in children, with a prevalence of 5% to 10%.^[2]^ The disease is most common in children aged 1 to 5 years.^[3]^ If left untreated, PN can result in childhood malnutrition, body wasting, rickets, anemia, and a decline in immune function, all adverse consequences that can affect growth and development both physically and intellectually, in addition to mental illness.^[4–5]^ In modern medicine, the main treatment for PN is drug treatment, including the use of supplementary trace elements to enhance peptin levels and gastric dynamics^[6]^ However, it is often difficult to determine the exact cause of anorexia in children.^[7]^ Due to the poor compliance of children on oral drugs and the high occurrence of side effects,^[8]^ their clinical application is limited.

As a painless and non-drug therapy, infantile massage is widely applicable to the pediatric disease spectrum, with the advantages of simple operation and a comfortable experience. In clinical practice, massage represents a dominant therapy for PN. Through many years of clinical practice, it has been shown that children with anorexia who receive massage therapy not only significantly improve their appetite, but relieve sallow complexion, abdominal distension, and other accompanying symptoms. Modern medical studies have shown that massage therapy can increase vagus nerve activity and promote appetite regulatory factors, thus increasing gastrointestinal peristalsis^[9]^ and improving digestive function. Massage intervention also correlates with improves the correlation between the brain and intestine axis, intestinal flora activity, and the regulation of host metabolism.

In this study, we explore the biological mechanism(s) of anorexia from the perspective of intestinal flora and the regulation of host metabolism. We further reveal the action site and therapeutic mechanism(s) of pediatric massage for the treatment of anorexia. This furthers our understanding of the optimal treatment methods for the treatment of PN in the clinic.

## 2 Methods

### 2.1 Study design

This single-blind, randomized controlled clinical study will follow the ethical principles of the Ministry of China's Ministry of Health “Ethical Review Measures for Biomedical Research Involving Human Beings (2007)”, the SFDA “Standard for Quality Management of Drug Clinical Trials (2003)”, “The Regulations on Clinical Trials of Medical Devices (2004)”, the WMA “Helsinki Declaration”, the CIOMS “International Ethical Guidelines for Biomedical Research on Human Beings”. The project was evaluated and approved by the Research Ethics Committee of Gansu Provincial Hospital of Traditional Chinese Medicine. (Ethical batch No. FJ/04-IRB/C/018-V3.0, Ethics Review Adoption Form places in the Supplemental Digital Content) Treatments are to be performed in the Gansu Provincial Hospital of Traditional Chinese Medicine from January 1, 2020 solstice and December 31, 2023. Arrangements for pre-intervention, intervention, and evaluation are shown in Supplementary Material 1. The flow chart of each stage of the study are shown in Supplementary Material 2.

### 2.2 Research subjects

All children meeting the eligibility criteria will be recruited through the Department of Pediatrics and Acupuncture and Massage at the Gansu Hospital of Traditional Chinese Medicine. At the time of admission, members of the research group will explain all relevant study content to the subjects and their families. Signed informed consent forms will be provided by all participants.

### 2.3 Inclusion criteria

All subjects will be required to meet the known diagnostic criteria for anorexia in children.^[1]^ The criteria include

(1) a refusal to eat adequate amounts of food for at least 1 month;
(2) a failure to communicate hunger and a lack of interest in food;
(3) significant growth deficiency;
(4) a refusal to eat that is unrelated to an underlying medical illness or a traumatic event;
(5) aged 2 to 12;
(6) agree to receive massage therapy;
(7) consent to the study either directly or through a legal guardian.

### 2.4 Exclusion criteria

(1) Patients with heart, brain, liver, kidney, systemic diseases, or acute infection;
(2) local skin breakage on the back or area of massage;
(3) anorexia due to other diseases (e.g., anorexia nervosa);
(4) receiving other treatments for anorexia in the past month;
(5) participation in other clinical trials.

### 2.5 Intervention measures

#### 2.5.1 Massage group

The manipulation method will be divided into 6 stages. The child will be placed in the supine position or held by the caregiver. The following procedures will then be performed:

### 2.6 Limb Massage

1. Massage will be performed using the thumb radial finger to the finger root (× 200 repetitions).
2. The palm and tip of the thumb will be used to rub the horizontal grain of the proximal interphalangeal joint of the index finger, middle finger, ring finger, and little finger (× 100 repetitions).
3. Kneading of the thenar will be performed with the thumb-end (× 100 repetitions).
4. The PC8 (located in the center of the horizontal grain of the palm, in the middle of the second and third metacarpal bones) will be used as the center of the circle and rubbed clockwise with the thumb abdominal (× 100 repetitions).
5. The ST 36 (located at the lower margin of the patella) will be pressed to 3 inches in the depression of the lateral patellar ligament by transversing the finger next to the anterior tibial ridge with low levels of force using the thumb or a threaded surface (× 150 repetitions).

### 2.7 Proximal massage

1. Spine pinching: will be performed from the tip of coccyx on the midline of the back to the spinous process of the seventh cervical vertebra, through pinching and lifting 3 times along the spine (× 7 repetitions);
2. abdomen kneading: will be performed by massaging the abdomen clockwise with the palms (× 100 repetitions).

#### 2.7.1 Control group

Jianweixiaoshi tablets (national medicine approval Z36021464) will be administered as follows:

(1) aged 2 to 6 years: 2 tablets/time;
(2) aged 7 to 12 years: 3 tablets/time with chewing 3 times/d.

#### 2.7.2 Blank group

No interventions will be performed.

### 2.8 Randomized grouping

A total of 60 healthy children will be randomly selected for physical examinations. A statistical expert not involved in the design of the study will use SPSS 22.0 software to generate a series of random sequences, which will be marked onto a card and placed in a sealed envelope. Each subject will receive an envelope at a time-point consistent with the test conditions. Subjects will be randomly divided based on their number into control or test groups (n = 60 per group). For each child, the chiropractor will open the envelope to obtain the grouping code.

### 2.9 Single-Blinding

The massage doctor will have no prior knowledge of the grouping information or treatment regimen contained in the envelope. All participants and relevant health care personnel will not interfere with the study. The evaluator will not be aware of the treatment protocol received by each subject and instructed family members will be asked to complete all relevant study scales. Statisticians will be blinded during all statistical analysis.

### 2.10 End of study/discontinuation

The study will be discontinued when the following complications are encountered:

(1) serious adverse events or specific physiological changes;
(2) voluntarily discontinuation;
(3) poor compliance, including a failure to cooperate or comply with treatment after repeated explanations by the clinician;
(4) receiving food or alternative treatments for anorexia;
(5) results of those exceeding half of the total course of treatment available for inclusion in the statistical analysis of treatments.

### 2.11 Outcomes

#### 2.11.1 Main Outcomes

##### 2.11.1.1 Structure of the intestinal flora

Feces samples from 3 groups of children will be collected and stored at −80 °C. Total genomic DNA will be extracted from all feces samples using commercial DNA purification kits as per the manufacturer's recommendations. Samples will be stored at −20 °C after qualitative detection. Concentrations will be determined through 0.8% agar-gel electrophoresis. For the detection of the 16S rRNA V6 region, the upstream primer 967F (CNACGCGAAGAACCTTANC) and downstream primer 1046R (CGACAGCCATGCANCACCT) will be used for all amplification reactions. The amplification system was 25uL, Takara Ex Taq was used, and the template was 2 uL. Amplification conditions will be as follows: 94 °C for 2 minutes; 94 °C for 30 seconds; 57 °C for 30 seconds; 72 °C for 30 seconds (× 30 cycles); final extension of 72 °C for 5 minutes.

#### 2.11.2 Short chain fatty acids *(*SCFAs*)* assays

The levels of SCFAs in the feces of each group will be determined using the GC method. Briefly, 0.5 g of each sample will be added to 4 mL of diluent, consisting of 15 mL of 100 mmol/L 2-ethyl butyrate and 50 mL of 5 mmol/Ld hydrochloride through spiral blending. Samples will be centrifuged at 4000 r/min for 10 minutes, and 1 mL subjected to ether extraction 3 times. It will then be added to internal standard solutions (10 μL) and right amount of anhydrous sodium sulfate, and the ether will be transferred to a small bottle containing a plug in a ventilated environment to aid evaporation. Samples will be covered and stored at 4 °C prior to testing. According to the standard curves of each acid, the content of SCFAs in each sample will be calculated from meteorological chromatographic charts.

#### 2.11.3 Secondary outcomes

Serum trace element levels, including Zn, Fe, and Ca will be measured by ICP-AES. Hemoglobin levels will be determined using resistive methods. Excretion rates of D-xylose in the urine will be determined using the reduction method. Gastric emptying and motility will be assessed using Color Doppler Ultrasonography.

#### 2.11.4 Complications and adverse events

All adverse events will be monitored during the assigned intervention and throughout the study period. Adverse events related to massage and oral medication, such as flatulence, abdominal pain, and increased bowel motility due to medication will be reported to the appropriate regulatory authority.

#### 2.11.5 Data management

All data will be recorded independently by 2 trained research assistants in the form of case report forms. All information will be kept strictly confidential. Since the known risks of massage are minimal, formal data monitoring committees are not required for data collection. The study will be regularly monitored and reviewed by independent investigators within the hospital staff.

#### 2.11.6 Statistical analysis

SPSS22.0 statistical software will be used for all analysis. Data will be graded using rank sum tests. *χ*^2^ tests will be used for data counts. Measurement data will be shown as the mean ± standard deviation. Data with a normal distribution will be analyzed using a 1-way analysis of variance. Least Significant Difference tests will be adopted for parity of variance. A Games–Howell test will be adopted for data with a non-normal distribution. The rank sum test was used for those who did not conform to normal distribution. *P* < .05 will be considered statistically significant.

## 3 Discussion

The intestinal flora forms a large and complex micro ecosystem that plays an important role in the maintenance of human health, including the normal function of the gastrointestinal tract.^[10]^ During childhood, disturbances in the intestinal flora can occur due to the relatively immature micro ecological environment,^[11]^ leading to effects on the neuroendocrine system that lead to a loss of appetite and food intake,^[12]^ increasing the risk of anorexia. The main metabolite of intestinal flora are SCFAs.^[13]^ SCFAs specifically bind to G-protein-coupled receptors in the intestinal tract^[14]^ to activate endocrine cells to secrete appetite-suppressing hormones, leading to a loss of appetite.^[15]^ Approximately 5% of SCFAs are excreted in the feces^[16]^ providing a relatively reliable and stable detection index for anorexia.

Massaging is a medical method to prevent and cure diseases of specific limb movements in the human body. Massaging is non-invasive, easy to obtain cooperation, and shows high safety. It also shows good application prospects for alternative drug therapy in children with anorexia, and has been shown to be effective for alleviating functional digestive associated diseases.^[17–19]^ The traditional Chinese massage theory suggests that both distal treatment of the meridian of the limbs and proximal treatment (local treatment such as kneading the spine and abdomen) can restore gastrointestinal function. The effects of distal treatment result from its influence on the autonomic nervous system and childhood psychology. By massaging the limbs, the peripheral blood flow is increased, the autonomic nervous system is activated, and the gastrointestinal parasympathetic nerve is excited.^[20]^ During massage, comfortable physical contact can also relieve anxiety and shorten the psychological distance between massage doctors and children.^[21]^ The mechanism of proximal treatment is mainly physical, by stimulating local acupoints and their meridional locations, the mechanical power of manipulation can be used to directly affect gastrointestinal muscle groups, thereby stimulating intestinal smooth muscle, promoting peristaltic function, reducing gastric residual volume and improving digestion.^[22]^ Continuous stimulation through massage can transform mechanical energy into heat energy, promoting intestinal blood circulation and alleviating gastrointestinal dynamic abnormalities. However, a lack of relevant research on the regulatory mechanisms of intestinal flora by massage therapy exist. In this study, we will provide new evidence for the regulation of intestinal flora in response to massage.

## 4 Advantages of the study

1. Massage therapy, as a painless and non-drug natural therapy, shows good safety and efficacy, and is easily accepted by children.
2. The detection of intestinal flora and SCFAs by 16S rRNA and GC allows the therapeutic mechanism(s) of massage on the treatment of anorexia in children to be objective and data-based.
3. As the main indicator, fecal tests are more acceptable to children than blood tests.

## 5 Limitations of the study

1. The control group drug will receive a widely used clinical drug that is recommended by Chinese guidelines. Its safety and efficacy have been proven through years of clinical application, but it is not currently recommended in relevant international guidelines.
2. All subjects are of the same ethnicity. Whether massage therapy is effective for other ethnicities/regions requires confirmation in future multi-center and large-sample trials.
3. Intestinal flora are susceptible to a variety of factors, particularly dietary factors.^[23]^ It is therefore difficult to exclude the influence of diet on the digestive tracts of the children. This study will provide dietary and water guidance, providing suggestions to minimize the bias in the final results.

## Author contributions

**Conceptualization:** Hanyuan Gao, Xutong Zhang, and Hanteng Yang.

**Data collection and collation:** Xutong Zhang and Wenjie He.

**Formal analysis:** Xia zhao.

**Funding acquisition:** Hanyuan Gao and Xia Zhao.

**Methodology:** Hanyuan Gao, Xutong Zhang, and Shencai Li.

**Therapists:** Juan Han and Dongmei Li.

**Validation:** Shengcai Li and Hanteng Yang.

**Writing – original draft:** Hanyuan Gao, Xutong Zhang, and Wenjie He.

## Supplementary Material

Supplemental Digital Content

## Supplementary Material

Supplemental Digital Content

## Supplementary Material

Supplemental Digital Content
