# Supplementary material for: To study the intervention mechanism of pediatric massage on intestinal flora and host metabolism in children with anorexia
Source: Medicine (Baltimore). 2020 Nov 20;99(47):e23349. doi: 10.1097/MD.0000000000023349 (PMC7676532; doi:10.1097/MD.0000000000023349)
Supplement: Supplemental Digital Content [file medi-99-e23349-s001.pdf]

附件 4 伦理审查批件（FJ/04-IRB/C/018-V3.0）

伦理审查批件

批件号	2020-066-01		
项目名称	研究小儿推拿对厌食症患儿肠道菌群及宿主代谢的干预机制		
项目来源	国家自然科学基金		
研究单位	甘肃省中医院		
主要研究者	高汉媛		
审查类别	初始审查	审查方式	会议审查
审查日期	2020.5.8	审查地点	甘肃省中医院东院区主楼 15 楼中会议室
审查委员	米登海、王海东、罗向霞、杨维建、张定华、徐柏林、盛丽、刘庆龙、吴心音。		
批准文件	科研项目伦理审查申请表；主要研究者简历；研究经济利益声明；毕业证；执业证；GCP 证书；方案（版本号：1.0 版，版本日期：2020 年 4 月 29 日）；知情同意书（版本号：1.0 版，版本日期：2020 年 4 月 29 日）；任务书；研究者手册（版本号：1.0 版，版本日期：2020 年 4 月 29 日）；招募广告；研究病历。		

审查意见

根据《涉及人的生物医学研究伦理审查办法》（2016 年）、《药物临床试验伦理审查工作指导原则》（2010 年）、《药物临床试验质量管理规范》（2003 年）、《医疗器械临床试验质量管理规范》（2016 年）、WMA 《赫尔辛基宣言》 和 CIOMS 《人体生物医学研究国际道德指南》的伦理原则，经本伦理委员会审查，同意按所批准的临床研究方案、知情同意书等开展本项试验/研究。

请遵循伦理委员会批准的方案开展临床研究，保护受试者的健康与权利。

研究开始前，请申请人完成临床试验注册。

研究过程中若变更主要研究者，对临床研究方案、知情同意书等的任何修改，请申请人提交修正案审查申请。

发生严重不良事件，请申请人及时提交严重不良事件报告。

请按照伦理委员会规定的年度跟踪审查频率，申请人在截止日期前 1 个月提交研究进展报告；申请人应当向组长单位伦理委员会提交各中心研究进展的汇总报告；当出现任何可能显著影响试验进行或增加受试者危险的情况时，请申请人及时向伦理委员会提交书面报告。

研究纳入了不符合纳入标准或排除标准的受试者，符合终止试验规定而未让受试者退出研究，给予错误治疗或剂量，给予方案禁止的合并用药等没有遵从方案开展研究的情况；或可能对受试者的权益/健康以及研究的科学性造成不良影响等违背 GCP 原则的情况，请申请人提交违背方案报告。

申请人暂停或提前终止临床研究，请及时提交暂停/终止研究报告。

完成临床研究，请申请人提交结题报告。

跟踪说明	您的主审委员是盛丽，电话：15002591663。主审委员要按照跟踪审查频率进行跟踪，请接受。
年度跟踪审查频率	12 个月
有效期	从 2020.5.10 到 2021.5.9
联系人与联系电话	李玉梅，0931—2687005

伦理委员会主任委员签字	[illegible]
伦理委员会	甘肃省中医院伦理委员会 （盖章）
日期	2020.5.9

甘肃省中医院伦理委员会
