# Supplementary material for: To study the intervention mechanism of pediatric massage on intestinal flora and host metabolism in children with anorexia
Source: Medicine (Baltimore). 2020 Nov 20;99(47):e23349. doi: 10.1097/MD.0000000000023349 (PMC7676532; doi:10.1097/MD.0000000000023349)
Supplement: Supplemental Digital Content [file medi-99-e23349-s002.pptx]

## Slide 1
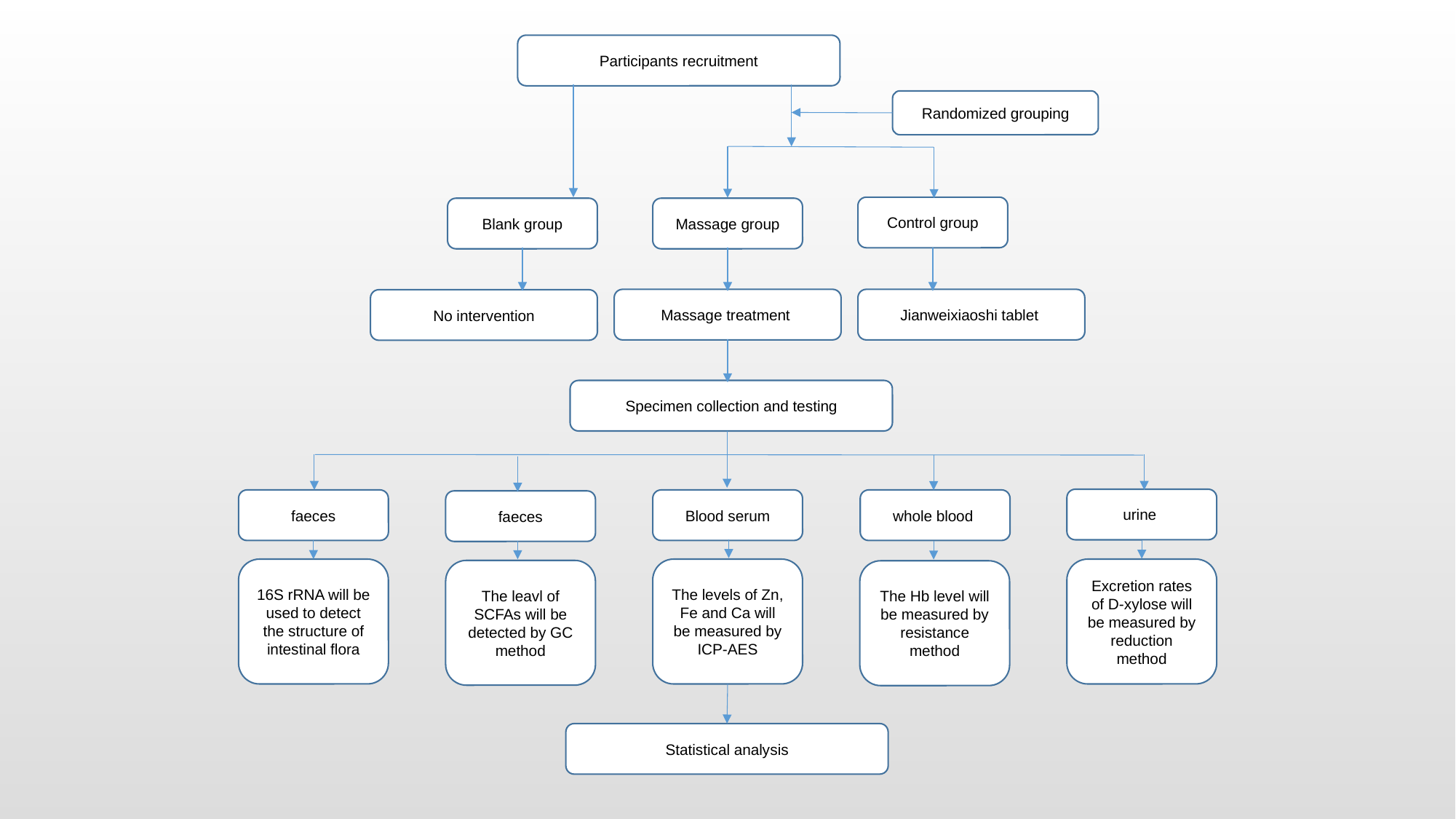

Participants recruitment
Randomized grouping
Control group
Blank group
Massage group
Massage treatment
Jianweixiaoshi tablet
No intervention
Specimen collection and testing
urine
faeces
whole blood
Blood serum
faeces
16S rRNA will be used to detect the structure of intestinal flora
Excretion rates of D-xylose will be measured by reduction method
The levels of Zn, Fe and Ca will be measured by ICP-AES
The leavl of SCFAs will be detected by GC method
The Hb level will be measured by resistance method
Statistical analysis
