# Supplementary material for: To study the intervention mechanism of pediatric massage on intestinal flora and host metabolism in children with anorexia
Source: Medicine (Baltimore). 2020 Nov 20;99(47):e23349. doi: 10.1097/MD.0000000000023349 (PMC7676532; doi:10.1097/MD.0000000000023349)
Supplement: Supplemental Digital Content [file medi-99-e23349-s003.doc]

| The schedule of trial enrolment,interventions and assessments | | | |
| --- | --- | --- | --- |
|  | **Enrolment** | **Study period** | |
|  | **Pre-intervention** | **Intervention** | **Evaluation procedures** |
| **Enrolment:** |  |  |  |
| **Assessment of eligibility** | **•** |  |  |
| **Informed consent** | **•** |  |  |
| **Randomisation** | **•** |  |  |
| **Interventions:** |  |  |  |
| **Massage** |  | **•** |  |
| **Jianweixiaoshi tablets** |  | **•** |  |
| **No interventions** |  |  |  |
| **Assessments:** |  |  |  |
| **Structure of the intestinal flora** | **•** |  | **•** |
| **The levels of SCFAs** | **•** |  | **•** |
| **Serum trace element levels** | **•** |  | **•** |
| **Hb levels** | **•** |  | **•** |
| **Excretion rates of D-xylose** | **•** |  | **•** |
| **Complications and adverse events:** |  | **•** | **•** |

SCFAs，Short chain Fatty Acids；Hb，Hemoglobin；
